# Luminescence and photoelectrochemical properties of size-selected aqueous copper-doped Ag–In–S quantum dots†

**DOI:** 10.1039/c8ra00257f

**Published:** 2018-02-16

**Authors:** Alexandra Raevskaya, Oksana Rozovik, Anastasiya Novikova, Oleksandr Selyshchev, Oleksandr Stroyuk, Volodymyr Dzhagan, Irina Goryacheva, Nikolai Gaponik, Dietrich R. T. Zahn, Alexander Eychmüller

**Affiliations:** L. V. Pysarzhevsky Institute of Physical Chemistry, National Academy of Sciences of Ukraine Kyiv 03028 Ukraine alstroyuk@ukr.net; Physical Chemistry, TU Dresden 01062 Dresden Germany oleksandr.stroyuk@chemie.tu-dresden.de; Saratov State University 410012 Saratov Russian Federation; Semiconductor Physics, Chemnitz University of Technology 09107 Chemnitz Germany; V. E. Lashkaryov Institute of Semiconductors Physics, National Academy of Sciences of Ukraine Kyiv 03028 Ukraine

## Abstract

Ternary luminescent copper and silver indium sulfide quantum dots (QDs) can be an attractive alternative to cadmium and lead chalcogenide QDs. The optical properties of Cu–In–S and Ag–In–S (AIS) QDs vary over a broad range depending on the QD composition and size. The implementation of ternary QDs as emitters in bio-sensing applications can be boosted by the development of mild and reproducible syntheses directly in aqueous solutions as well as the methods of shifting the photoluminescence (PL) bands of such QDs as far as possible into the near IR spectral range. In the present work, the copper-doping of aqueous non-stoichiometric AIS QDs was found to result in a red shift of the PL band maximum from around 630 nm to ∼780 nm and PL quenching. The deposition of a ZnS shell results in PL intensity recovery with the highest quantum yield of 15%, with almost not change in the PL band position, opposite to the undoped AIS QDs. Size-selective precipitation using 2-propanol as a non-solvent allows discrimination of up to 9 fractions of Cu-doped AIS/ZnS QDs with the average sizes in the fractions varying from around 3 to 2 nm and smaller and with reasonably the same composition irrespective of the QD size. The decrease of the average QD size results in a blue PL shift yielding a series of bright luminophors with the emission color varies from deep-red to bluish-green and the PL efficiency increases from 11% for the first fraction to up to 58% for the smallest Cu-doped AIS/ZnS QDs. The rate constant of the radiative recombination of the size-selected Cu-doped AIS/ZnS QDs revealed a steady growth with the QD size decrease as a result of the size-dependent enhancement of the spatial exciton confinement. The copper doping was found to result in an enhancement of the photoelectrochemical activity of CAIS/ZnS QDs introduced as spectral sensitizers of mesoporous titania photoanodes of liquid-junction solar cells.

## Abstract 2

Colloidal size-selected copper-doped Ag–In–S quantum dots were produced directly in aqueous solutions by fractionation/redispersion with a plethora of emission colors and a top luminescence quantum yield of around 60%.

## Graphical abstract

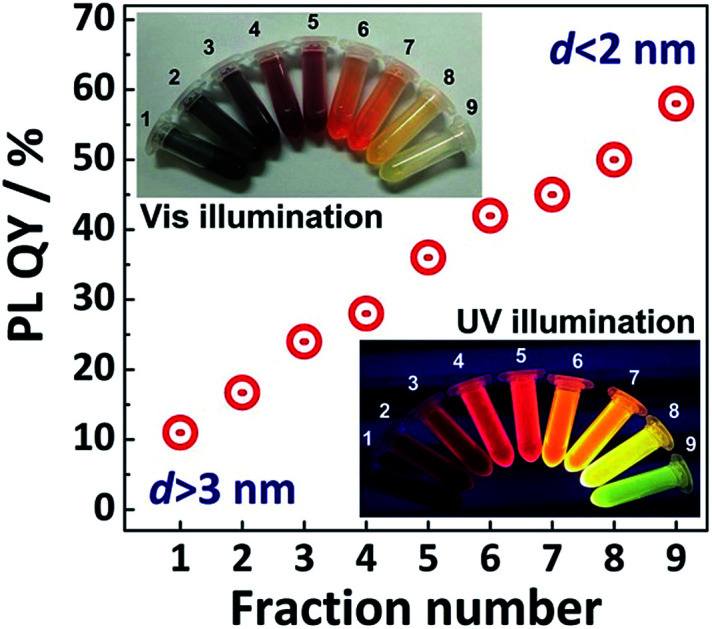

## Introduction

Metal chalcogenide nanocrystals with a size smaller than the doubled Bohr exciton radius, or quantum dots (QDs), reveal in many cases a unique combination of intense light absorbance, photoluminescence (PL) emission with high quantum yields (QYs), and a strong dependence of the bandgap (*E*_g_) and the energies of the charge carriers on the QD size. This allows for a broad variation of spectral, photophysical, and photochemical properties of such QDs by tailoring their size at a constant chemical composition. These properties of the metal-chalcogenide QDs might be introduced into a variety of light-harvesting and light-emitting applications, in particular, into photovoltaics, luminescent bio-labeling, light-emitting diodes, light detectors and concentrators, *etc.*^1–5^ Typically, the research on metal-chalcogenide QDs is focused on cadmium (CdX, X = S, Se, Te) and lead chalcogenides (PbX) capable of intense light absorption in the visible (CdX) and near-infrared (PbX) ranges and intensely emitting with narrow PL bands with PL QYs as high as 90–95%.^4–6^

However, the toxicity of CdX/PbX QDs and the products of their corrosion stimulates a search for alternative compounds with similar spectral characteristics and strong size-dependences of the electronic properties, in particular, among ternary and more complex chalcogenides of In, Ga, Sn, *etc.*^4,7–16^ This search focused on ternary sulfides CuInS_2_ (CIS) and AgInS_2_ (AIS) having narrow bandgaps of 1.5 eV and 1.85 eV, respectively^7–9,13,15^ and being well suited for solar light harvesting. Also, CIS and AIS QDs can emit strong PL with the spectral parameters broadly varying with the CIS/AIS QD composition, size, doping, *etc.*^7,8,11–15^ Such ternary compounds revealed a number of quite unique properties differing drastically from those of the binary CdX/PbX compounds, in particular, the immensely broad deviations from the stoichiometry while preserving the crystal lattice symmetry and quality, the capability to form a plethora of solid solutions *via* a partial substitution of sulfur with Se or Te, In – with Ga, and by alloying CIS (AIS) with ZnS, as well as the lattice preservation at a heavy doping with “alien” metal cations.^7,9–16^ These special features open many options for tailoring the band structure of the ternary compounds with related consequences as to the spectral sensitivity range, the charge carriers energies, the conductivity and the carrier mobility, *etc.*, which are unattainable for the binary compounds. This unprecedented variability of properties becomes even broader in the nanometer crystal size range, where size dependences of the electronic properties become expressed distinctly.^9–16^

Typically, the CIS (AIS) QDs are produced by “classic” heating up/hot injection approaches, developed in detail for CdX/PbX QDs, when the nucleation and ripening of the QDs occur in the high-boiling solvents playing simultaneously the role of capping ligands (such as trioctylphosphine oxide or oleylamine) or/and as sulfur source (1-dodecanethiol).^1,4,5,17–21^ These methods allow for a precise control over the QD size, size distribution and phase composition. However, many applications favor (like *e.g.* photovoltaics) or even demand (*e.g.* luminescent bio-labeling) the post-synthesis transfer of CIS (AIS) QDs into water. Conventionally, the transfer is performed by introducing bifunctional molecules, such as mercaptoacids, simultaneously being capable to bind strongly to the QD surface *via* the mercapto-group and to stabilize the QDs in water *via* the deprotonation of the carboxyl group providing an electrostatic shield against inter-particle interactions.^3,4,7,9,11–16,22^

To avoid such a two-step synthetic procedure and make the total synthesis more environmentally friendly the approaches to the synthesis of CIS (AIS) QDs directly in aqueous solutions have received recently an ever-growing attention.^3,4,10,23–26^ The research in this area has already yielded synthetic protocols for the preparation of strongly absorbing QD “inks” for the utilization as light-harvesting components of solar cells. However, the PL QYs of CIS (AIS) QDs produced directly in water still remain low and a further exploration is needed to improve the control over the QD size, surface chemistry and lattice perfection in aqueous syntheses of ternary QDs.

Recently we have reported on the aqueous synthesis and size selection of brightly luminescent AIS and core/shell AIS/ZnS QDs stabilized by mercaptoacetate (MA) anions.^27^ The size-selected AIS/ZnS QDs emit light with relatively high QYs of 45–46% for the best fractions, however, the spectral PL range is limited to around 700 nm (in terms of the PL band maximum position).

At the same time, many applications, such as light-emitting devices and luminescent bio-sensing in the first transparency window of the human skin require luminophors emitting in the near IR range. To extend the spectral range of the emission doping with Cu or alloying between AIS and CIS is proposed frequently.^14,15,28^ Cu doping was also found to be an efficient method of improving the charge carrier mobility in metal-chalcogenide QDs, in particular in AIS QDs, making them more efficient light harvesters in third-generation solar cells.^29–31^ Incorporation of copper ions into alloyed ZnS–In_2_S_3_ (ref. 32 and 33) and Zn–In–Se^34^ nanocrystals was found to result in a spectacular enhancement of the photocatalytic properties and PL efficiency, respectively. Cu doping of AIS and AIS/ZnS QDs synthesized by the heating-up method results in a shift of the emission to lower energies and was used to produce efficient luminescent markers for the imaging of tumor cells.^23,35^

In the present paper we summarize our studies on Cu-doped AIS (CAIS) and CAIS/ZnS QDs synthesized in aqueous solutions and subjected to post-synthetic size selection. Our results indicate that the Cu doping is an attractive method for tailoring the PL range allowing to shift the PL band down to 800 nm and still have a reasonably high PL QY. The size selection was found to be a potent tool of influencing both the spectral PL parameters and the PL efficiency, yielding CAIS/ZnS QDs with PL QYs reaching almost 60%.

## Materials and methods

### Chemicals

Indium(iii) chloride, silver and copper(ii) nitrates, zinc(ii) acetate dihydrate, Na_2_S·9H_2_O, NH_4_OH (aqueous 5.0 M solution), mercaptoacetic acid (MAA), 2-propanol were purchased from Sigma-Aldrich and Acros Organics and used without additional purification. All the solutions were prepared using deionized (DI) Milli-Q water (Millipore).

### Synthesis and size-selective precipitation of QDs

Aqueous colloidal solutions of AIS and CAIS QDs were prepared by a reaction between sodium sulfide and a mixture of silver(i) or silver(i) + copper(ii) and indium(iii) MA complexes similarly to our other reports.^27,36^ In a typical synthesis, the feed molar ratio of silver to indium to sulfur, Ag : In : S, was adjusted to 2 : 7 : 10. 2.0 mL aqueous 0.1 M AgNO_3_ solution, an aliquot (for example, 0.2 mL) of aqueous 0.1 M Cu(NO_3_)_2_ solution, and 2 mL aqueous 1.0 M MAA solution were added to 94 mL water under magnetic stirring and ambient conditions. The resulting turbid yellowish suspension becomes transparent after the addition of 0.45 mL aqueous 5.0 M NH_4_OH solution and colorless – after the addition of 0.7 mL aqueous 1.0 M InCl_3_ solution containing 0.2 M HNO_3_. Then, 1.0 mL aqueous 1.0 M Na_2_S solution was added at stirring and the resulting solution heated in a water bath at 90–95 °C for 30 min. The CAIS QDs were covered with a ZnS shell *via* the decomposition of Zn^II^–MA complex. For this, 2.0 mL aqueous 1.0 M MAA solution, 2.5 mL aqueous 1.0 M Zn(CH_3_COO)_2_ solution (with 0.01 M HNO_3_), and 0.3 mL aqueous 5 M NH_4_OH solution were added at intense stirring to 100 mL of the core QD solution and the mixture was additionally heated for 30 min.

The as-prepared CAIS/ZnS QD colloids were concentrated by a factor of ∼10 on a rotary evaporator at around 40 °C and denoted further as “crude” colloids. In a typical size-selection procedure, to 10 mL of crude colloids 2.5 mL of 2-propanol were added to initiate aggregation of the QDs with subsequent centrifugation at 4500 rpm for 5 min. The precipitate was separated and designated further as fraction #1. This procedure was repeated several times with fresh amounts (0.5 mL for each fraction) of 2-propanol producing fractions #2–9 till the complete exhaustion of QDs in the original crude colloid. Further details of the size-selective precipitation can be found in ref. 27. The collected precipitates #1–9 were each separately dissolved in 1 mL of Milli-Q water and stored in the dark at room temperature. For absorption and PL measurements the fractionated solutions were diluted by a factor of 10–500 (depending on the fraction number) by Milli-Q water.

### Characterization

Absorption and PL spectra were recorded using a UV-vis spectrophotometer Cary 60 and a fluorescence spectrometer Fluoromax 4, respectively, in standard 10.0 mm optical quartz cuvettes. The PL was excited at *λ* = 420 nm. The PL spectra were normalized to the optical density of the solutions at the excitation wavelength.

The PL QY was determined using AIS/ZnS QDs as a luminescence standard with an absolute QY of 37% measured using a Fluorolog 3 spectrometer equipped with a Quanta *Φ* integrating setup.^18^ The kinetic curves of the PL decays were registered for CAIS/ZnS colloids using a Horiba Jobin Yvon Fluorocube-01-NL. The samples were placed in a 10.0 mm quartz optical cuvette and excited with a 350 nm pulse of a NanoLED-350 diode (Horiba Jobin Yvon).

Details on the investigations by X-ray photoelectron spectroscopy (XPS), X-ray diffraction and Raman spectroscopy are provided in (ESI†).

The photoelectrochemical measurements were performed in a three-electrode cell with FTO/TiO_2_/QD heterostructures as photoanodes, a Pt counter-electrode, and an Ag/AgCl reference electrode. The details of the photoanode preparation and measurements are presented elsewhere.^37^

## Results and discussion

### Characterization of CAIS and CAIS/ZnS QDs

Colloidal CAIS and CAIS/ZnS QDs are characterized by an average hydrodynamic size of ∼4 nm and ∼4.2 nm, respectively (ESI, Fig. S1†), with no larger formations present indicating the individual character of each QD in the colloidal ensemble.

**Fig. 1:**
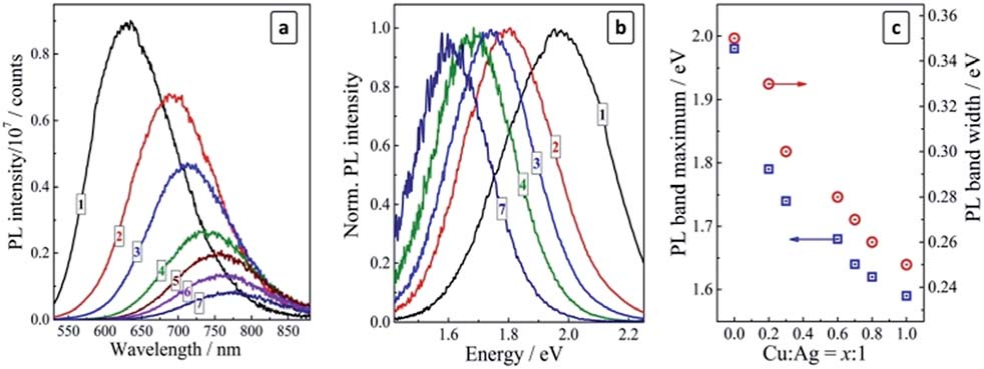
Original (a) and normalized (b) PL spectra of crude colloids of undoped AIS QDs (curve 1) and CAIS QDs prepared at Cu : Ag = 0.2 : 1 (curve 2), 0.3 : 1 (curve 3), 0.6 : 1 (curve 4), 0.7 : 1 (curve 5), 0.8 : 1 (curve 6), and 1 : 1 (curve 7). (c) Maximum position (blue squares) and spectral width (red circles) of the PL band of CAIS/ZnS QDs produced at a different Cu : Ag ratio. Ag : In : S = 1 : 7 : 10.

**Fig. 2:**
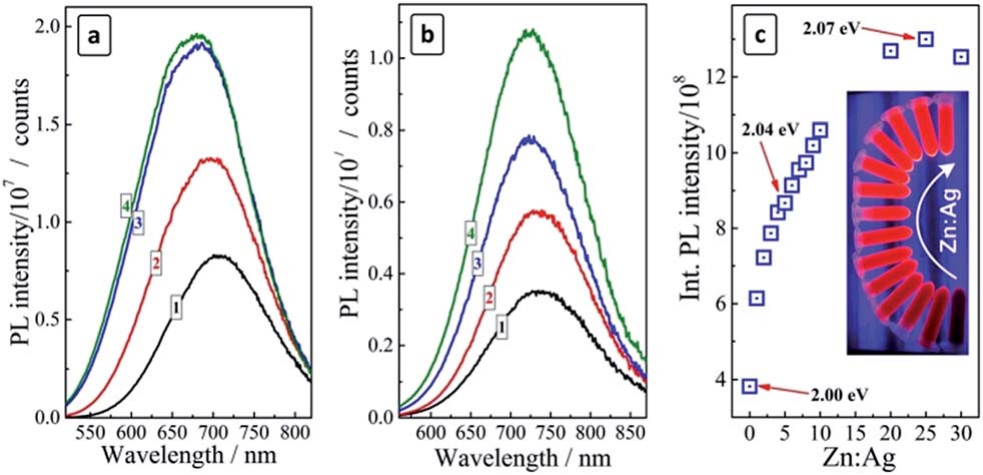
(a and b) PL spectra of AIS QDs (a) and CAIS QDs (b) before Zn addition (curves 1) and after the addition at a Zn : Ag ratio of 2 : 1 (curves 2), 10 : 1 (curves 3), and 20 : 1 (curves 4); (c) integral PL intensity of CAIS/ZnS QDs as a function of Zn : Ag ratio. Figures on the graph indicate bandgap values for corresponding samples. Insert: photograph of CAIS/ZnS series with an increasing Zn : Ag ratio under the UV illumination. Ag : In : S = 2 : 7 : 10.

The undoped AIS QDs (prepared at Ag : In : S = 1 : 7 : 10) exhibit a broad PL band centered at 630 nm (1.97 eV) with a Stokes shift of about 0.4 eV and a spectral width of ∼0.35 eV (Fig. 1a and b, curves 1). An increase in the silver content to Ag : In : S = 2 : 7 : 10 results in a red shift of the PL band maximum to around 700 nm (Fig. 2a, curve 1).

As the copper ions are introduced to AIS QDs the PL band shifts to longer wavelengths and gradually loses in intensity (Fig. 1a). Both the red shift and the PL decrease are proportional to the molar Cu : Ag ratio set at the synthesis. An XPS study (see more details on XPS measurements below) of two selected samples, corresponding to Cu : Ag = 1 : 10 and 5 : 10, showed that CAIS QDs purified by the precipitation with 2-propanol and redispersed in DI water, reveal atomic Cu-to-Ag ratios of (1–2) : 10 and (4–5) : 10, respectively. Therefore, the copper dopant content in the CAIS QDs is very close to the total amount of copper introduced at the synthesis.

For the ultimate case of Cu : Ag : In : S = 1 : 1 : 7 : 10 the PL band maximum can be found at around 780 nm (∼1.6 eV), while the PL intensity is reduced by a factor of about 15 (Fig. 1a, curve 7). The evolution of the PL band from a “yellow” one (centered at around 2.0 eV) to a “red” one (peaked at ∼1.6 eV) can be visualized by the normalized PL spectra presented in Fig. 1b as well as by the dependence of the PL band maximum energy on the copper-to-silver ratio (Fig. 1c, blue squares).

It was reported for the copper-diffused AIS QDs that Cu can fill the inherent vacancies in the AIS lattice as well to partially substitute silver ions in their lattice sites.^30,35^ The filling of vacancies should be reflected in a narrowing of the distribution of possible electron states participating in the radiative recombination and, hence, in a narrowing of the PL band. Such a narrowing is indeed observed in the present case: the spectral width of the PL band decreases from 0.35 eV for the pristine AIS QDs to 0.25 eV for the heavily doped CAIS QDs prepared at Cu : Ag = 1 : 1 (Fig. 1c, red circles) indicating that copper does indeed fill the vacancies in the silver–indium–sulfide lattice. A similar narrowing is also observed after the incorporation of Zn^2+^ ions into the AIS lattice.^17,19,23,27,35^

The deposition of a ZnS shell on the surface of both AIS and CAIS QDs was found to result in a strong PL enhancement (Fig. 2a and b), however, the character of the changes of spectral parameters of the PL band is somewhat different between undoped and doped QDs. As the amount of ZnS is elevated, the PL band of AIS QDs increases in intensity and shows a “blue shift” (Fig. 2a).

These observations indicate the passivation of the surface defect states participating in non-radiative electron–hole recombination and the diffusion of Zn^2+^ ions into the lattice of AIS QDs resulting in an increment of the bandgap and the lattice constant.^17,18,23,26,27,38^ In the case of CAIS QDs only a slight blue shift of the PL band is observed along with a PL enhancement (Fig. 2b), supporting the above assumption about a partial filling of the AIS lattice vacancies by copper ions that inhibit the subsequent diffusion of Zn^2+^. The PL intensity increases by a factor of more than 3 as the ZnS shell is deposited and the Zn : Ag ratio changed from zero to 20–25 (Fig. 2c). The further increase of the ZnS content is not accompanied by a further PL intensity increase, rather resulting in a certain decrease of the emission intensity. The highest PL QY obtained for Zn : Ag = 25 was 15%.

### Size selection of CAIS QDs

Recently, we have shown the feasibility of size-selective precipitation of aqueous MA-stabilized AIS and AIS/ZnS QDs by using 2-propanol as a “bad” solvent.^27^ The method is based on the addition of 2-propanol to an aqueous solution of AIS QDs resulting in the precipitation of a fraction of the largest QDs from the colloidal ensemble. The precipitate can be separated and redispersed in pure water forming a colloidal solution stable for months. The repetition of this procedure with an increasing amount of 2-propanol yields many (up to 10–11) fractions of size-selected QDs with distinctly different optical properties and an average size varying from around 2 to 3–3.5 nm.^27^ Here we show that a similar method can be successfully applied for the size selection of the doped MA-capped CAIS and CAIS/ZnS QDs.

**Fig. 3:**
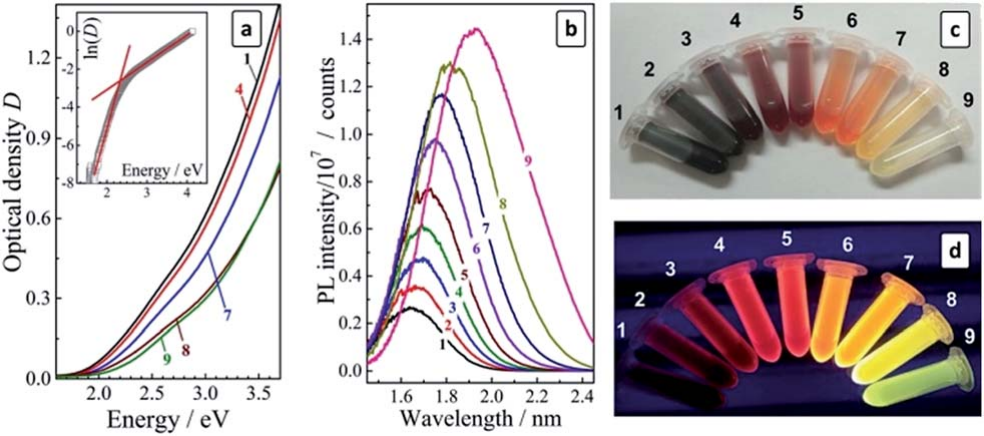
(a and b) Absorption (a) and PL (b) spectra of size-selected CAIS/ZnS QDs (the curve numbers correspond to the fraction numbers) PL was registered after a normalization of the QD concentration to the same optical density (∼0.1) at the PL excitation wavelength. Insert in (a): curve 9 in the coordinates “ln(*D*) − quantum energy”; (c and d) photographs of size-selected CAIS/ZnS QD colloids taken under ambient (c) and UV (d) illumination (365 nm).

Thus, by increasing the volumetric 2-propanol-to-water ratio from 0.3 to 3.3 up to 9 separate fractions of CAIS/ZnS QDs were discriminated from the original colloidal solution (Cu : Ag : In : S : Zn = 0.2 : 2 : 7 : 10 : 25) differing distinctly in their spectral characteristics. The color of the solutions varies from dark-brown for fraction #1 (the largest QDs) to bright-yellow for fraction #9 of the smallest QDs, while the emission color is changed from deep-red for fraction #1 to bluish-green for fraction #9 (see photographs in Fig. 3). As the PL QY and the stability against aggregation of the CAIS/ZnS QDs appeared to be much higher than those for CAIS QDs without protective shells we performed the following studies most exclusively with the core/shell CAIS/ZnS QDs synthesized at the core composition providing the highest PL QY (Cu : Ag : In : S = 0.2 : 2 : 7 : 10).

A TEM study of size-selected CAIS/ZnS QDs yielded only blurred images (ESI, Fig. S2†), most probably, due to the charging of the sample in the electron beam resulting in oscillations of the sample and compromising the beam focusing. This problem seem to be of a general character for the small aqueous AIS-based QDs observed by us earlier for undoped MA-capped AIS/ZnS^27^ and recently – for the glutathione-capped AIS/ZnS QDs.^39^ Nevertheless, it can be seem from the TEM images that the most populated fractions #1–2 of the present CAIS/ZnS QDs contain 3–4 nm particles (in accordance with the DLS measurements), while fractions #8–9 showed the presence of smaller QDs of ∼2 nm in size.

The absorption spectra of size-selected CAIS/ZnS QDs reveal continuous bands with no distinct maxima or humps and with the band edge shifting continuously to shorter wavelengths as the fraction number increases (Fig. 3a). We have shown earlier^27^ that the absorption band of AIS and AIS/ZnS QDs near the band edge is characterized by a relatively strong contribution of sub-bandgap states (Urbach absorption) masking the exact position of the band edge. Therefore, a satisfyingly precise determination of the bandgap from the absorption spectra cannot be achieved when the data is replotted in the coordinates of the Tauc equation. In the case of the size-selected series of AIS QDs the contribution of the Urbach absorption increased with decreasing QD size resulting in large uncertainties in the *E*_g_ values. We proposed to evaluate the bandgap as the crosspoint of two linear sections of the absorption spectra replotted in the coordinates of the Urbach equation, one of them corresponding to the sub-bandgap absorption and the other – to the interband electron transitions, as shown in Fig. 3a (insert). The values of these bandgaps derived for different fractions using the Urbach equation, *E*^U^_g_, are collected in Table 1.

**Table 1:** Some parameters of absorption/PL and metal ratios for a series of size-selected CAIS/ZnS QDs (starting composition Cu : Ag : In : S : Zn = 0.2 : 2 : 7 : 10 : 2)

Fraction no.	*E* ^U^_g_, eV	*E*_PL_, eV	Δ*S*, eV	FWHM, eV	PL QY, %	In : Ag	Zn : Ag	C : Ag	Cu : Ag	Cu : Zn
1	2.05	1.64	0.41	0.31	11	1.8	1.1	0.7	0.11	0.10
2	2.08	1.66	0.42	0.31	17	1.7	1.0	0.7	0.13	0.13
3	2.10	1.68	0.42	0.31	24	2.1	1.0	1.0	0.14	0.14
4	2.12	1.69	0.43	0.32	28	2.2	1.0	1.0	0.08	0.08
5	2.13	1.71	0.42	0.35	36	2.2	1.0	1.3	0.08	0.10
6	2.14	1.75	0.39	0.38	42	3.0	1.2	1.8	0.11	0.09
7	2.17	1.78	0.39	0.39	45	4.7	1.3	3.0	0.12	0.09
8	2.23	1.82	0.41	0.41	50	5.0	1.7	3.4	0.22	0.13
9	2.32	1.92	0.40	0.50	58	11.0	3.0	7.5	0.27	0.09

The bandgap of the size-selected CAIS/ZnS QDs was found to expand from 2.05 eV to 2.32 eV as the fraction number increased from #1 to #9. The energy of the PL band maximum (*E*_PL_) of the size-selected QDs increases as well, shifting from 1.64 eV to 1.92 eV. Consequently, the Stokes shift, Δ*S* = *E*^U^_g_ − *E*_PL_, remains reasonably constant varying slightly around 0.4 eV (Table 1), justifying further the employed method of the bandgap evaluation.

The composition of the size-selected series of the CAIS/ZnS QDs prepared at Cu : Ag : In : S : Zn = 0.2 : 2 : 7 : 10 : 2 was analyzed by XPS (second part of Table 1). The atomic Cu : Ag ratio was found to vary around 0.1 without sharp deviations matching the original Cu : Ag ratio set during the QD synthesis. The Cu : Zn ratio was found to be rather stable as well, varying around 0.1 for fractions #1–7 as expected from the above stoichiometry. These results indicate that the size-selected CAIS/ZnS QDs are characterized by a more or less similar content of the copper dopant throughout the entire series from #1 to #7. The last two fractions #8 and #9 reveal some deviations from the above tendencies but the amount of QDs in these fractions was very small (by two orders of magnitude smaller than, for example, in fractions #1 and #2) which may compromise the accuracy of the XPS measurements.

The ratio of indium to silver was found to increase from around 2 for fraction #1–5 to ∼5 for fractions #7, 8 and increasing sharply for fraction #9 with the smallest CAIS/ZnS QDs (Table 1). In most cases this ratio differs from the preset value indicating that a portion of the In^3+^ ions is bound to MA and remains unprecipitated in the supernatant solution as well as forms a protective complex ligand layer on the surface of QDs. As shown above, the relative amount of sulfur increases almost linearly with a decrease in the QD size supporting this conclusion. Also, the carbon-to-silver ratio increases showing the same tendency as the In : Ag ratio further supporting the assumption on the partial binding of In^3+^ ions to the surface complexes with MAA. The Zn : Ag ratio keeps reasonably stable for fractions #1–7 indicating that size-selected QDs are characterized by a uniform ZnS shell in different fractions. Some increase in the Zn : Ag ratio for the two last fractions can also be an indication that a portion of Zn(ii) binds to MA forming surface protective complexes.

**Fig. 4:**
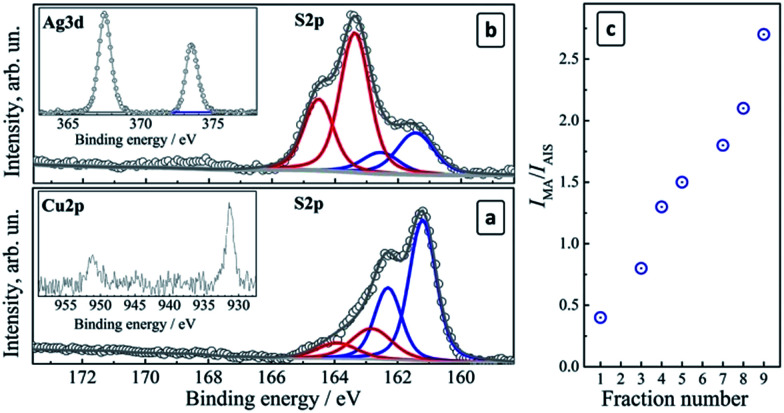
(a and b) XPS spectra of CAIS/ZnS QDs in the S 2p range for fractions #2 (a) and #7 (b). Inserts: (a) Cu 2p range, (b) Ag 3d range; (c) *I*_MA_/*I*_AIS_ ratio as a function of the QD fraction number.

The electron binding energies for In 3d, Ag 3d, and Cu 2p derived from XPS spectra are essentially identical for the fractions #1–9. The In 3d range (not shown here) shows a doublet at 442.2 eV/451.7 eV typical for In(iii) in ternary copper/silver indium sulfides.^40^ The Cu 2p range (Fig. 4a, insert) exhibits doublet peaks at 931.6 eV/951.5 eV for all the studied samples typical for Cu(i) in CIS compounds.^29,40^ This indicates that the Cu(ii) ions introduced during the synthesis of the crude colloidal solutions are reduced to Cu(i) by MAA at the moment of the complex formation as previously observed for aqueous CIS QDs.^41^ A doublet at 367.5 eV/373.5 eV in the Ag 3d range (Fig. 4b, inset) is typical for Ag(i) in binary and ternary metal chalcogenides.^40^

The high-resolution XPS spectra of size-selected CAIS QDs in the S 2p range showed a complex band that can be deconvoluted into two (fraction #2, Fig. 4a) or three (fraction #7, Fig. 4b) doublets separated by a characteristic spin–orbit splitting of 1.2 eV. The first two doublets at 161.2 eV/162.4 eV and 163.4 eV/164.6 eV were assigned to sulfur in the metal–sulfide AIS lattice (*S*^2−^) and to the surface-adsorbed MA species, respectively.^40^

As the fraction number increases (and the QD size gradually diminishes), the intensity of the lattice sulfur signal (*I*_AIS_) becomes weaker while the MA sulfur signal intensity (*I*_MA_) increases (compare Fig. 4a and b). The intensity ratio *I*_MA_/*I*_AIS_ was found to increase in an almost linear way with the increase of the fraction number (Fig. 4c). If a similar surface density of MA ligands is assumed for different QD sizes, this observation may indicate a decrease in the QD size in the consecutive fractions, resulting in an increase in the total surface area of colloidal QDs and in the amount of the surface ligand species as compared to the lattice sulfur atoms. The surface MA concentration depends on the total QD surface area and is proportional to *d*^2^ (*d* is the QD size), while the amount of the lattice sulfur increases as *d*^3^ and therefore the *I*_MA_/*I*_AIS_ ratio should be inversely proportional to the QD size (as 1/*d*) and proportional to the fraction number, in accordance with the observations presented in Fig. 4c.

**Fig. 5:**
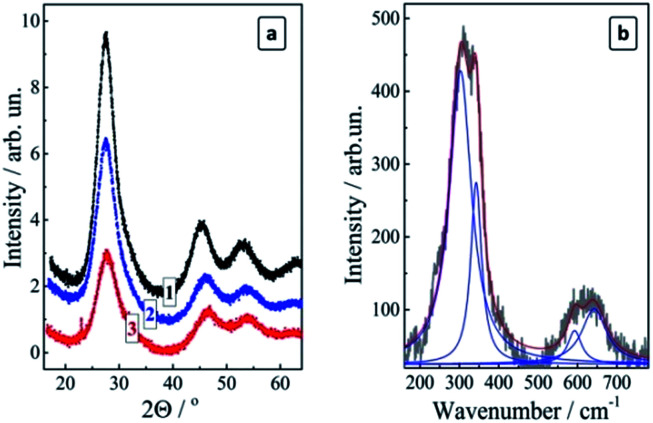
X-ray diffractograms (a) and Raman spectrum (b) of the size-selected CAIS/ZnS QDs. Cu : Ag : In : S : Zn = 0.2 : 2 : 7 : 10 : 2, (a) fraction #1 (curve 1), #5 (curve 2), and #9 (curve 3), (b) fraction #3. Blue and red lines in (b) – results of fitting with Lorentz profiles.

The X-ray diffractograms of the size-selected CAIS/ZnS QDs reveal diffraction patterns typical for the chalcopyrite silver–indium–sulfide compounds for all studied fractions (Fig. 5a). The main (100) peak at 27.5° is slightly shifted to larger angles (smaller lattice parameters) with respect to pure AIS QDs (26.9° (ref. 27)) indicating that the Cu doping decreases the lattice parameter of the CAIS QDs because of a smaller ionic radius of Cu^+^ as compared to Ag^+^.^29^ The relative intensity of the diffraction peaks decreases somewhat with an increase of the fraction number as a result of an increased disordering in smaller QDs, in accordance with the below-discussed PL observations. The size of the coherent X-ray diffraction domain (close to the average QD size) was estimated by the Scherrer equation to be 2.8 nm (fraction #1), 2.0 nm (fraction #5), and 1.6 nm (fraction #9). This range is very similar to the size range of the non-doped size-selected MA-capped AIS QDs studied by us earlier.^27^ The similarity indicates that the size-selective precipitation produced a more or less identical series of the size-selected QD fractions in both cases provided for the same 2-propanol/water ratio.

A study of the complete set of the size-selected fractions #1–9 of CAIS/ZnS with Raman spectroscopy was obstructed by the ever-increasing PL background resulting in meaningful spectral features only for the fractions #1–3. The spectra were found to be identical for all three fractions under two different excitation wavelengths (488 and 515 nm) and revealed a broadened feature in the range of 240–380 cm^−1^ with an overtone above 500 cm^−1^ (Fig. 5b). The main feature can be deconvoluted into two peaks at around 303 cm^−1^ and 343 cm^−1^, which is characteristic for AIS compounds.^29^ No other admixture phases were detected in the Raman spectra.

Similarly to the parental colloidal solutions, the size-selected CAIS/ZnS QDs revealed broad PL bands with the intensity increasing strongly with a decrease of the QD size (Table 1). The PL QY was found to increase in a roughly linear manner from 11% for fraction #1 up to 58% for fraction #9 of the smallest CAIS/ZnS QDs. Fig. 3c and d show that the size selection yields a set of quite intensely emitting luminophors with the emission color varying from deep red for the starting fractions to light-yellow and yellow-green for the least populated but the brightest fractions #8 and #9.

Typically, PL QYs between ∼20% and ∼40% are reported for luminescent AIS/ZnS QDs with the inner AIS core doped with Zn^2+^ ions.^17,19,23,26,38^ A careful optimization of the Ag-to-In ratio in AIS QDs and the AIS-to-ZnS ratio in alloyed ZAIS QDs allowed to reach PL QYs of 60–80%.^18,20,25^ One of the highest PL QY of 87% was recently observed for “double-shell” ZAIS/ZnIn_2_S_4_/ZnS heterostructured QDs.^21^ These figures show that the luminescent size-selected CAIS/ZnS QDs discussed in the present paper are quite competitive with the best reported AIS-based nano-luminophors and, most probably, reveal the highest PL QY reported to date for the copper-doped AIS QDs.

The spectral width of the PL band was found to increase considerably with a decrease in the size of CAIS/ZnS QDs, from 0.31 eV for fraction #1 up to 0.50 eV for the fraction #9 of the smallest QDs (Table 1). The same tendency was earlier observed by us for the size-selected undoped AIS/ZnS QDs^27^ and accounted for by an increase disordering of the QDs of decreasing size and a broadening of the spectrum of electron and hole states participating in the radiative recombination. This explanation is corroborated by an increase of the characteristic Urbach energy deduced from the sub-bandgap “tails” of the corresponding absorption spectra.^27^

The dynamics of the radiative recombination in the size-selected CAIS/ZnS QDs was studied by time-resolved PL spectroscopy for fractions #1–7 which have almost the same composition as shown by the XPS measurements. The kinetic curves of the PL decays of CAIS/ZnS QDs revealed a distinctly non-monoexponential character typical for ternary QDs. For the fractions of AIS/ZnS and CAIS/ZnS QDs produced at the same volumetric ratio of 2-propanol to water the PL was found to decay faster for the doped QDs as compared with the undoped ones. For example, the radiative lifetime *τ* of the QDs was estimated as the time when the original PL intensity decreases by a factor of *e*. It was found to decrease from 405 ns for AIS/ZnS (fraction #3) to 355 ns for CAIS/ZnS QDs (fraction #3).

This observation along with the reduction in the PL QY induced by copper doping indicates a higher rate of the non-radiative recombination in the doped CAIS/ZnS QDs as compared with AIS/ZnS. An opposite trend was earlier reported for surface-doped AIS/ZnS QDs,^35^ where the PL lifetime increased as a result of Cu doping. This report along with the present results indicate a crucial role of copper localization, when the relative probability of the Cu ions participation in radiative and non-radiative recombination depends on the mode of copper introduction producing opposite effects for surface-implanted Cu ions as in ref. 35 and for Cu ions introduced on the stage of QD nucleation, as in the present work.

**Fig. 6:**
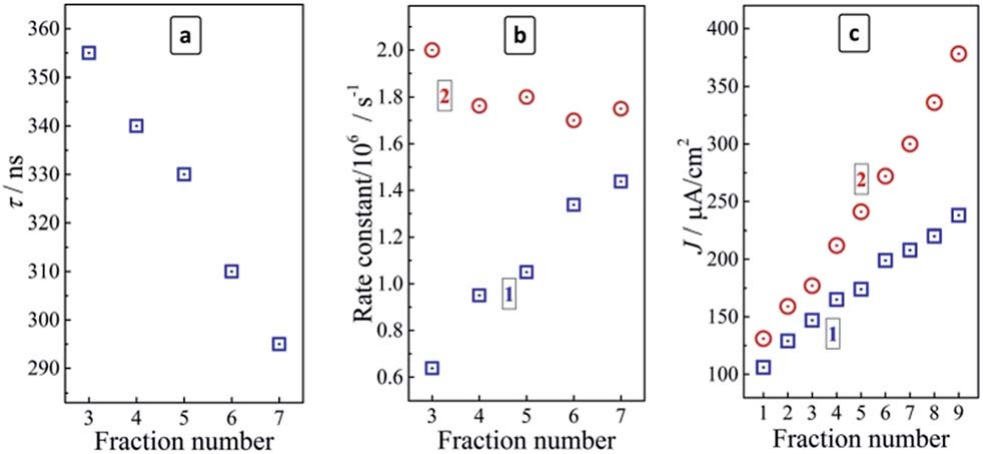
(a and b) Average PL lifetime (a), and rate constants (b) of radiative (blue squares 1) and non-radiative recombination (red circles 2) a series of the size-selected colloidal CAIS/ZnS QDs. (c) Photocurrent density in the three-electrode photoelectrochemical cells based on FTO/TiO_2_/CAIS photoanodes with the size-selected AIS/ZnS (blue squares 1) and CAIS/ZnS QDs (red circles 2).

As shown above by the stationary PL measurements the negative influence of Cu doping on the PL intensity and rate becomes counterbalanced by a decrease in the average size of CAIS/ZnS QDs, resulting in a strong enhancement of the radiative-recombination. The analysis of the stationary PL spectra of the size-selected QDs revealed a steady broadening of the PL band with a size decrease indicative of a broader spectrum of states participating in the PL emission. Therefore, it can be expected that the emission becomes faster, which is corroborated by the experimental data indicating a reduction in the radiative life time from 355 ns to 295 ns as the fraction number increases from 3 to 7 (Fig. 6a).

By combining data on the stationary PL QY and the average PL lifetimes the rate constants of both radiative and non-radiative recombination in the size-selected CAIS/ZnS QDs, *k*_r_ and *k*_nr_, can be derived by well-reported approaches^31^ as functions of the QD size (or fraction number). The *k*_r_ value was found to grow steadily with a QD size decrease, increasing from 0.63 × 10^6^ to 1.44 × 10^6^ s^−1^ as the fraction number increases from 3 to 9 (Fig. 6b). At the same time, *k*_nr_ was found to be relatively constant, varying between 2.00 × 10^6^ s^−1^ for fraction #3 and 1.63 × 10^6^ s^−1^ for fraction #9, that is by less than 20% of the average value, which is too small to assess it as a distinct tendency for a decrease. Therefore, the non-radiative recombination rate constant can be accepted as unchanged in the studied QD size range. The increase of *k*_r_ observed for the size-selected CAIS/ZnS QDs with roughly the same composition, Cu dopant content, ZnS shell thickness and defect density (the latter derived from unchanged *k*_nr_) may, therefore, be ascribed to the effect of increased charge carrier confinement favoring the radiative electron–hole recombination. As suggested by the above-discussed XPS data, the amount of In and Zn complexes with MA adsorbed on the QD surface increases with a QD size decrease and the more dense ligand shell can also have some contribution into the observed enhancement of the PL efficiency with the increasing fraction number.

In our earlier studies we have found that the PL efficiency of CIS/ZnS QDs correlates with the photoelectrochemical activity of TiO_2_/CIS nanoheterostructures produced from the same core CIS QDs but without the passivating ZnS shell (or with a very thin one).^37^ In the present work we have investigated the photoelectrochemical activity of TiO_2_/QD composites based on the size-selected CAIS/ZnS QDs introduced as photoanodes in three-electrode cells with a Pt counter-electrode and aqueous Na_2_S/Na_2_S_*x*_ electrolyte, similar to ref. 37.

The concentration of all the fractions of CAIS/ZnS colloids used for the preparation of the photoanodes was normalized to an equal optical density at 350 nm, that is, far from the absorption band edge, where the absorbance can be assumed to be proportional to the QD concentration and a possible size-dependence of the molar extinction coefficient ignored as a first approximation. In this way, the TiO_2_/QD photoanodes were characterized by roughly the same QD volume density.

It was found that the copper doping enhances the photoelectrochemical activity of AIS QDs since the TiO_2_/CAIS/ZnS photoanodes produce higher photocurrent densities for all the studied fractions as compared to the undoped TiO_2_/AIS/ZnS analogues (Fig. 6c). The doping-induced photoactivity increment reveals a dependence on the QD size increasing from 24% for the largest QDs (fraction #1) to ∼60% for the smallest CAIS/ZnS QDs (fraction #9). The photocurrent density produced by TiO_2_/CAIS/ZnS heterostructures correlates well with the PL QY of the individual colloidal CAIS/ZnS (Table 1) indicating that the PL measurements can be used as a diagnostic tool to assess and predict the light-harvesting efficiency of the QDs introduced into the heterostructured photoanodes of the liquid-junction solar cells.

## Conclusions

The copper-doping of aqueous non-stoichiometric AIS QDs was found to result in a red shift of the PL band maximum from around 630 nm to ∼780 nm and a PL quenching. The deposition of a ZnS shell onto doped CAIS QDs allows to recover the relatively intense emission with a highest PL QY of 15% (at a molar Cu : Ag : In : S : Zn ratio of 0.2 : 2 : 7 : 10 : 25) while almost not changing the position of the PL band. The stability of the PL band position in the presence of the protective ZnS shells in the case of CAIS QDs was attributed to the filling of inherent vacancies in the AIS lattice by Cu(i) ions impeding the subsequent inclusion of Zn(ii) ions from the shell.

Size-selective precipitation using 2-propanol as a non-solvent allows to discriminate up to 9 fractions of CAIS/ZnS QDs from the original ensemble revealing distinctly different optical properties. The average size of QDs in the fractions changes from about 3 to 2 nm and smaller similar to the earlier reported case of undoped AIS and AIS/ZnS QDs.^27^ Studies of the fractionated colloids by X-ray photoelectron and Raman spectroscopy showed that the fractions contain only the AIS-like chalcopyrite phase without other phase admixtures.

The decrease of the average size of the CAIS/ZnS QDs results in a blue shift of the PL maximum yielding a series of relatively brightly emitting luminophors with the emission color varying from deep-red to bluish-green and the PL QY increasing from 11% for the first fraction up to 58% to the smallest CAIS/ZnS QDs in the least populated fraction #9.

The rate constant of radiative electron–hole recombination *k*_r_ showed a steady increase from 0.63 × 10^6^ to 1.44 × 10^6^ s^−1^ with the QD size decrease while the value of the non-radiative recombination rate varied only slightly with the QD fraction number increasing from 3 to 7. The enhancement of the radiative electron–hole recombination was attributed to the size-dependent spatial exciton confinement in the CAIS/ZnS QDs.

The copper doping was found to result in an enhancement of the photoelectrochemical activity of CAIS/ZnS QDs introduced as spectral sensitizers of mesoporous titania photoanodes of liquid-junction solar cells. The doping-induced photoactivity increment increases from 24% for the largest QDs to ∼60% for the smallest QDs, the photocurrent density correlating closely with the PL QY of original colloidal CAIS/ZnS QDs.

## Conflicts of interest

There are no conflicts to declare.

## Supplementary Material

RA-008-C8RA00257F-s001

## References

[cit1] Rogach A. L., Eychmüller A., Hickey S. G., Kershaw S. V. (2007). Small.

[cit2] Rogach A. L., Gaponik N., Lupton J. M., Bertoni C., Gallardo D. E., Dunn S., Li Pira N., Paderi M., Repetto P., Romanov S. G., O'Dwyer C., Sotomayor Torres C. M., Eychmüller A. (2008). Angew. Chem., Int. Ed..

[cit3] Lesnyak V., Gaponik N., Eychmüller A. (2013). Chem. Soc. Rev..

[cit4] Jing L., Kershaw S. V., Li Y., Huang X., Li Y., Rogach A. L., Gao M. (2016). Chem. Rev..

[cit5] Zu G., Zeng S., Zhang B., Swihart M. T., Yong K. T., Prasad P. N. (2016). Chem. Rev..

[cit6] Gaponik N., Hickey S. G., Dorfs D., Rogach A. L., Eychmüller A. (2010). Small.

[cit7] Thomas S. R., Chen C. W., Date M., Wang Y. C., Tsai H. W., Wang Z. M., Chueh Y. L. (2016). RSC Adv..

[cit8] Azimi H., Hou Y., Brabec C. J. (2014). Energy Environ. Sci..

[cit9] Fan F. J., Wu L., Yu S. H. (2014). Energy Environ. Sci..

[cit10] Sandroni M., Wegner K. D., Aldakov D., Reiss P. (2017). ACS Energy Lett..

[cit11] Regulacio M. D., Han M. Y. (2016). Acc. Chem. Res..

[cit12] Kershaw S. V., Susha A. S., Rogach A. L. (2013). Chem. Soc. Rev..

[cit13] Kolny-Olesiak J., Weller H. (2013). ACS Appl. Mater. Interfaces.

[cit14] Knowles K. E., Hartstein K. H., Kilburn T. B., Marchioro A., Nelson H. D., Whitham P. J., Gamelin D. R. (2016). Chem. Rev..

[cit15] Coughlan C., Ibáñez M., Dobrozhan O., Singh A., Cabot A., Ryan K. M. (2017). Chem. Rev..

[cit16] Reiss P., Carrière M., Lincheneau C., Vaure L., Tamang S. (2016). Chem. Rev..

[cit17] Tang X., Ho W. B. A., Xue J. M. (2012). J. Phys. Chem. C.

[cit18] Kameyama T., Takahashi T., Machida T., Kamiya Y., Yamamoto T., Kuwabata S., Torimoto T. (2015). J. Phys. Chem. C.

[cit19] Gabka G., Bujak P., Giedyk K., Ostrowski A., Malinowska K., Herbich J., Golec B., Wielgus I., Pron A. (2014). Inorg. Chem..

[cit20] Xiang W., Xie C., Wang J., Zhong J., Liang X., Yang H., Luo L., Chen Z. (2014). J. Alloys Compd..

[cit21] Ko M., Yoon H. C., Yoo H., Oh J. H., Yang H., Do Y. R. (2017). Adv. Funct. Mater..

[cit22] Torimoto T., Kameyama T., Kuwabata S. (2014). J. Phys. Chem. Lett..

[cit23] Deng D., Cao J., Qu L., Achilefu S., Gu Y. (2013). Phys. Chem. Chem. Phys..

[cit24] Xiong W. W., Yang G. H., Wu X. C., Zhu J. J. (2013). J. Mater. Chem. B.

[cit25] Wang L., Kang X., Pan D. (2016). Phys. Chem. Chem. Phys..

[cit26] Song J., Ma C., Zhang W., Li X., Zhang W., Wu R., Cheng X., Ali A., Yang M., Zhu L., Xia R., Xu X. (2016). ACS Appl. Mater. Interfaces.

[cit27] Raevskaya A., Lesnyak V., Haubold D., Dzhagan V., Stroyuk O., Gaponik N., Zahn D. R. T., Eychmüller A. (2017). J. Phys. Chem. C.

[cit28] Pradhan N., Adhikari S. D., Nag A., Sarma D. D. (2017). Angew. Chem., Int. Ed..

[cit29] Dasgupta U., Saha S. K., Pal A. J. (2014). Sol. Energy Mater. Sol. Cells.

[cit30] Guchhait A., Pal A. J. (2013). ACS Appl. Mater. Interfaces.

[cit31] Saha S. K., Guchhait A., Pal A. J. (2014). Phys. Chem. Chem. Phys..

[cit32] Li F., Chen G., Luo J., Huang Q., Luo Y., Meng Q., Li D. (2013). Catal. Sci. Technol..

[cit33] Shen S., Zhao L., Zhou Z., Guo L. (2008). J. Phys. Chem. C.

[cit34] Ke J., Li X., Zhao Q., Shi Y., Chen G. (2014). Nanoscale.

[cit35] Chen S., Demillo V., Lu M., Zhu X. (2016). RSC Adv..

[cit36] Raevskaya A. E., Ivanchenko M. V., Stroyuk O. L., Kuchmiy S. Y., Plyusnin V. F. (2015). J. Nanopart. Res..

[cit37] Raevskaya A., Rosovik O., Kozytskiy A., Stroyuk O., Dzhagan V., Zahn D. R. T. (2016). RSC Adv..

[cit38] Torimoto T., Adachi T., Okazaki K., Sakuraoka M., Shibayama T., Ohtani B., Kudo A., Kuwabata S. (2007). J. Am. Chem. Soc..

[cit39] Stroyuk O., Raevskaya A., Spranger F., Selyshchev O., Dzhagan V., Schulze S., Zahn D. R. T., Eychmüller A. (2018). J. Phys. Chem. C.

[cit40] Naumkin A. V. , Kraut-Vass A. and Gaarenstroom S. W., NIST X-ray Photoelectron Spectroscopy Database, NIST, Stand. Ref. Database 20, Version 4.1, 2012

[cit41] Hamanaka Y., Ozawa K., Kuzuya T. (2014). J. Phys. Chem. C.

