# Correction: Luminescence and photoelectrochemical properties of size-selected aqueous copper-doped Ag–In–S quantum dots

**DOI:** 10.1039/d3ra90104a

**Published:** 2023-10-27

**Authors:** Alexandra Raevskaya, Oksana Rozovik, Anastasiya Novikova, Oleksandr Selyshchev, Oleksandr Stroyuk, Volodymyr Dzhagan, Irina Goryacheva, Nikolai Gaponik, Dietrich R. T. Zahn, Alexander Eychmüller

**Affiliations:** a L. V. Pysarzhevsky Institute of Physical Chemistry, National Academy of Sciences of Ukraine Kyiv 03028 Ukraine alstroyuk@ukr.net; b Physical Chemistry, TU Dresden 01062 Dresden Germany oleksandr.stroyuk@chemie.tu-dresden.de; c Saratov State University 410012 Saratov Russian Federation; d Semiconductor Physics, Chemnitz University of Technology 09107 Chemnitz Germany; e V. E. Lashkaryov Institute of Semiconductors Physics, National Academy of Sciences of Ukraine Kyiv 03028 Ukraine

## Abstract

Correction for ‘Luminescence and photoelectrochemical properties of size-selected aqueous copper-doped Ag–In–S quantum dots’ by Alexandra Raevskaya *et al.*, *RSC Adv.*, 2018, **8**, 7550–7557, https://doi.org/10.1039/C8RA00257F.

The authors regret that an incorrect version of Fig. 3 was included in the original article. The correct version of Fig. 3 is presented below.

**Fig. 3:**
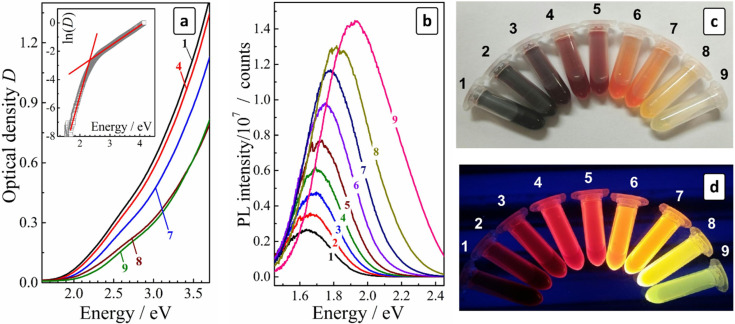
(a and b) Absorption (a) and PL (b) spectra of size-selected CAIS/ZnS QDs (the curve numbers correspond to the fraction numbers). PL was registered after normalization of the QD concentration to the same optical density (∼0.1) at the PL excitation wavelength. Insert in (a): curve 9 in the coordinates “ln(*D*) – quantum energy”; (c and d) photographs of size-selected CAIS/ZnS QD colloids taken under ambient (c) and UV (d) illumination (360–370 nm).

The Royal Society of Chemistry apologises for these errors and any consequent inconvenience to authors and readers.

## Supplementary Material

