# Supplementary material for: Luminescence and photoelectrochemical properties of size-selected aqueous copper-doped Ag–In–S quantum dots
Source: RSC Adv. 2018 Feb 16;8(14):7550–7. doi: 10.1039/c8ra00257f (PMC9078464; doi:10.1039/c8ra00257f)
Supplement: RA-008-C8RA00257F-s001 [file RA-008-C8RA00257F-s001.pdf]

Electronic Supplementary Material (ESI) for RSC Advances.
This journal is © The Royal Society of Chemistry 2018

Electronic Supplementary Information

Luminescence and Photoelectrochemical Properties of Size-Selected Aqueous Copper-Doped Ag-In-S Quantum Dots

Alexandra Raevskaya[a,b], Oksana Rozovik[a], Anastasiya Novikova[c], Oleksandr Selyshchev[d], Oleksandr Stroyuk*[a,b], Volodymyr Dzhagan[e], Irina Goryacheva[c], Nikolai Gaponik[b], Dietrich R. T. Zahn[d] and Alexander Eychmüller[b]

[a]L.V . Pysarzhevsky Institute of Physical Chemistry, National Academy of Sciences of Ukraine, Kyiv, 03028, Ukraine.

[b]Physical Chemistry, TU Dresden, 01062 Dresden, Germany.

[c]Saratov State University, 410012, Saratov, Russian Federation.

[d]Semiconductor Physics, Chemnitz University of Technology, 09107 Chemnitz, Germany

[e]V. E. Lashkaryov Institute of Semiconductors Physics, National Academy of Sciences of Ukraine, Kyiv, 03028, Ukraine.

Corresponding author:

**Dr. Oleksandr Stroyuk*, Physikalische Chemie, Technische Universität Dresden, Bergstraße 66b, 01062 Dresden, Germany, Tel. +49(0)351 463 34351; Laboratory of Organic Photovoltaics and Electrochemistry, L.V. Pysarzhevsky Institute of Physical Chemistry, National Academy of Sciences of Ukraine, Kyiv, Prosp. Nauky 31, 03028, Ukraine, Tel. +38(0) 44 525 02 70,

e-mail: alstroyuk@ukr.net; oleksandr.stroyuk@chemie.tu-dresden.de

Experimental details

X-ray photoelectron spectroscopy (XPS) experiments were performed with an ESCALAB™ 250Xi X-ray Photoelectron Spectrometer Microprobe (Thermo Scientific) for QD solutions deposited onto FTO and dried in a nitrogen flow. The monochromatized Al K_{α} ($h\nu$ = 1253.6 eV) X-ray source provided a spectral resolution of 0.5 eV. To prevent possible charging of the sample it was flooded with low kinetic energy electrons. Spectra deconvolution and quantification were performed using the Thermo ScientificTM Avantage Software.

X-ray diffraction patterns were collected using a Bruker D2 Phaser diffractometer in an angle range of 2Θ = 10–100° with a rate of 0.05° per min using monochromatized copper K_{α} irradiation. The samples were produced by dropcasting QD solutions mixed with acetone (1:1) on a polished silicon wafer that was also used as an internal standard for the evaluation of diffraction peak widths. The drop-cast solutions were dried at room temperature in a nitrogen flow.

Raman spectra were acquired by excitation with a 488 nm laser (Sapphire, Coherent) or a 515 nm laser (Cobolt) and registered with a spectral resolution of 2 cm^{-1} using a LabRam HR800 micro-Raman system equipped with the liquid nitrogen cooled CCD detector (Symphony). The incident laser power was kept below 0.02 mW, in order to avoid sample heating under the microscope objective (100×).

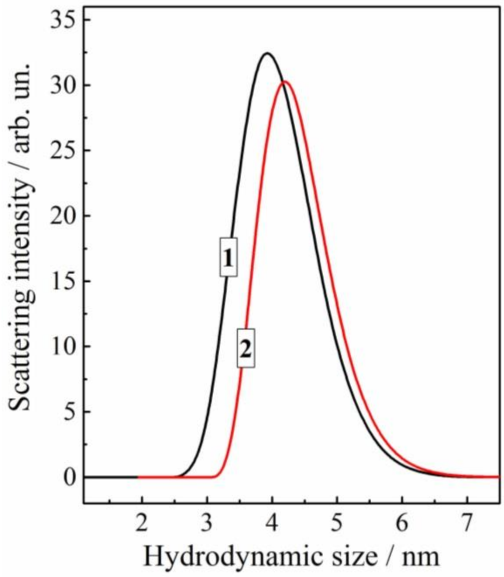

Figure S1. Hydrodynamic size distribution of colloidal CAIS QDs (curve 1) and CAIS/ZnS QDs (curve 2) determined by the dynamic light scattering (DLS). Cu:Ag:In:S = 0.2:2:7:10 (curve 1), Cu:Ag:In:S:Zn = 0.2:2:7:10:2 (curve 2). DLS measurements were performed with a Zetasizer Nano (Malvern Instruments).

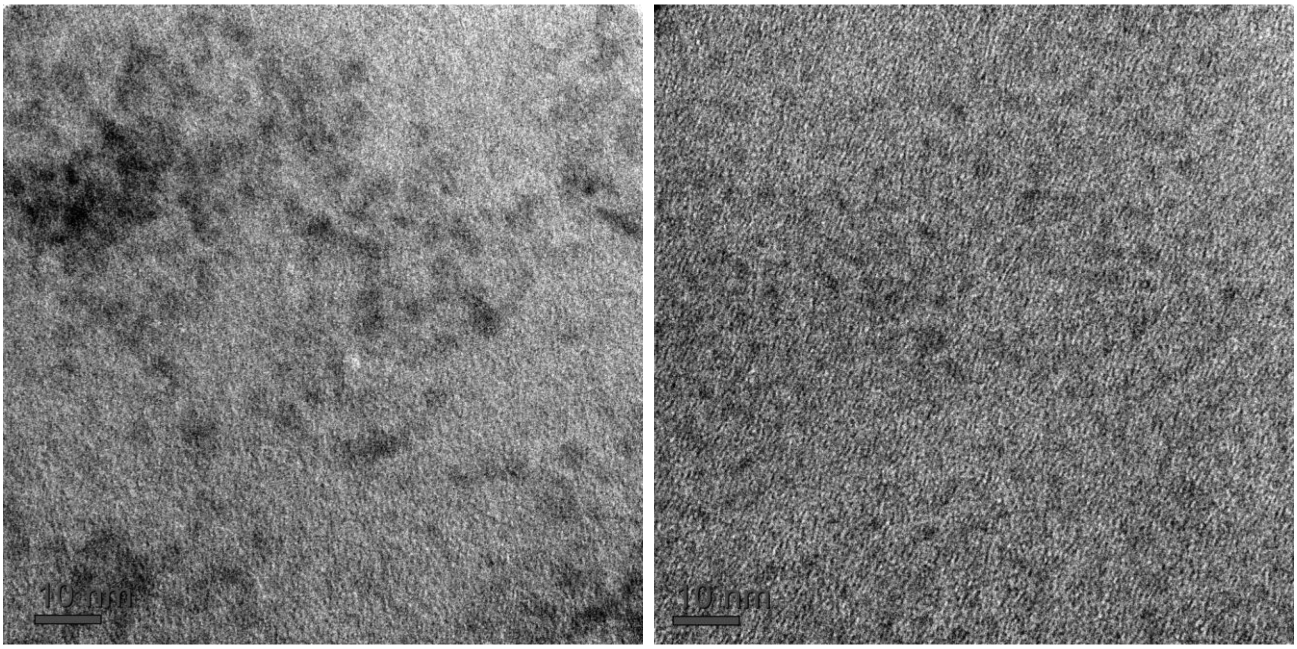

a **b**

Figure S2. (a) TEM images of size-selected CAIS/ZnS QDs from fractions #1–2 (a) and fractions #8–9 (b). Cu:Ag:In:S:Zn = 0.2:2:7:10:2. Transmission electron microscopy (TEM) measurements were performed using a FEI Tecnai G2 microscope at an accelerating voltage of 300 kV.
